# Crystal structure of a seven-substitution mutant of hydroxynitrile lyase from rubber tree

**DOI:** 10.1107/S2053230X25007034

**Published:** 2025-08-27

**Authors:** Colin T. Pierce, Lauren R. Greenberg, Meghan E. Walsh, Ke Shi, Drenen J. Magee, Hideki Aihara, Wendy Gordon, Robert L. Evans, Romas J. Kazlauskas

**Affiliations:** a Department of Biochemistry, Molecular Biology and Biophysics University of Minnesota Minneapolis Minnesota USA; b Crown College, Saint Bonifacius, Minnesota, USA; Baylor College of Medicine, Houston, USA

**Keywords:** engineered proteins, hydroxynitrile lyases, α/β-hydrolase fold, esterases

## Abstract

The α/β-hydrolase fold superfamily includes esterases and hydroxynitrile lyases which, despite catalyzing different reactions, share a Ser–His–Asp catalytic triad. We report a 1.99 Å resolution crystal structure of HNL6V, an engineered variant of hydroxynitrile lyase from *Hevea brasiliensis* (*Hb*HNL) containing seven amino-acid substitutions (T11G, E79H, C81L, H103V, N104A, G176S and K236M). The structure reveals that HNL6V maintains the characteristic α/β-hydrolase fold while exhibiting systematic shifts in backbone and catalytic atom positions. Compared with wild-type *Hb*HNL, the C^α^ positions in HNL6V differ by a mean of 0.2 ± 0.1 Å, representing a statistically significant displacement. Importantly, the catalytic triad and oxyanion-hole atoms have moved 0.2–0.8 Å closer to their corresponding positions in SABP2, although they remain 0.3–1.1 Å from fully achieving the configuration of SABP2. The substitutions also increase local flexibility, particularly in the lid domain covering the active site. This structural characterization demonstrates that targeted amino-acid substitutions can systematically shift catalytic geometries towards those of evolutionarily related enzymes.

## Abstract 2

The crystal structure of HNL6V, a seven-substitution variant of hydroxynitrile lyase from rubber tree, reveals that engineered mutations designed to mimic the homologous esterase SABP2 shift the catalytic atom positions intermediate between those of the parent enzyme and the target esterase.

## 1. Introduction

Hydroxynitrile lyases (HNLs) catalyze the enantioselective interconversion of cyanohydrins and aldehydes/ketones (Bracco *et al.*, 2016 ▸; Lanfranchi *et al.*, 2013 ▸; Dadashipour & Asano, 2011 ▸). HNLs occur in at least four protein folds; this paper focuses on HNLs with an α/β-hydrolase fold. This fold consists of eight β-strands connected by α-helices and contains a catalytic triad, typically Ser–His–Asp (Ollis *et al.*, 1992 ▸; Bauer *et al.*, 2020 ▸). One example of an HNL with an α/β-hydrolase fold is the hydroxynitrile lyase from the Pará rubber tree *Hevea brasiliensis* (*Hb*HNL).

Most enzymes in the α/β-hydrolase fold superfamily are esterases. HNLs in this superfamily, such as *Hb*HNL, diverged from esterases approximately 100 million years ago (Rauwerdink *et al.*, 2016 ▸; Devamani *et al.*, 2016 ▸). *Hb*HNL shares 44% sequence identity and 62% similarity over 260 positions with the modern esterase SABP2 (salicylic acid-binding protein 2 from tobacco; Supplementary Fig. S1). *Hb*HNL and SABP2 also use the same Ser–His–Asp catalytic triad even though the catalytic mechanisms of ester hydrolysis and cyanohydrin cleavage differ significantly (Zuegg *et al.*, 1999 ▸).

In this work, we report the structure of a seven-substitution variant of *Hb*HNL. Six of the seven substitutions replace residues in and around the active site with a corresponding residue from SABP2 (Thr11Gly, Glu79His, Cys81Leu, Asn104Ala, Gly176Ser and Lys236Met). The seventh substitution, His103Val, introduces an amino acid similar to the leucine in SABP2. A similar histidine-to-valine substitution in the homologous hydroxynitrile lyase from *Baliospermum montanum* stabilized the protein (Dadashipour *et al.*, 2011 ▸). Comparison of the catalytic atom positions shows that they have moved closer to the positions in SABP2 but still differ by 0.3–1.1 Å. The largest statistically significant differences are in the positions of the histidine N^ɛ2^ and aspartate O^δ2^.

## 2. Materials and methods

### 2.1. Protein production and purification

The gene encoding the wild-type HNL from *H. brasiliensis* in the pSE420 plasmid (Hasslacher *et al.*, 1996 ▸) was recloned into a pET-21a(+) plasmid (Table 1 ▸). The enzyme variant HNL6V was constructed via inverse PCR (Ochman *et al.*, 1988 ▸) using non-overlapping mutagenic primers in a sequential manner starting from HNL3V (Nedrud *et al.*, 2014 ▸). Briefly, mutagenic primers were annealed to the plasmid template in a back-to-back, outward-facing orientation and were amplified using New England Biolabs (NEB) Q5 HiFi polymerase (M0491S) to produce a linear, double-stranded DNA product containing the desired mutation(s). Primers were designed using the *NEBaseChanger* (https://nebasechanger.neb.com) web tool and checked for secondary structure and self-dimer/heterodimer propensity with the *OligoAnalyzer Tool* (https://www.idtdna.com/pages/tools/oligoanalyzer) from Integrated DNA Technologies (IDT), Coralville, Iowa, USA. Primers were purchased from IDT and used without further purification. PCR was performed using a Bio-Rad 2000 Thermal Cycler with the following conditions: initial denaturation at 98°C for 30 s, 30 cycles of denaturation (98°C for 30 s), annealing (calculated annealing temperature for 25 s) and extension (72°C for 150 s), and a final extension step of 72°C for 30 s. The PCR products were treated with a KLD enzyme mix (NEB M0554S), which phosphorylates the 5′ ends of the linearized PCR products, ligates the phosphorylated ends and degrades the original plasmid template. 5 µl of KLD product was used directly to transform chemically competent *Escherichia coli* DH5α cells (NEB C2988) according to the manufacturer’s protocol and plate them onto lysogeny broth (LB) plates containing 100 µg ml^−1^ carbenicillin. After overnight growth at 37°C, individual colonies were picked and grown overnight in LB medium and the plasmids were extracted using a NEB Monarch Plasmid mini-prep kit (NEB T1010). Plasmid concentrations were measured spectrophoto­metrically at 260 nm using a NanoDrop 2000 (Thermo Scientific) and diluted to <1.0 OD units if necessary. Sanger sequencing from Genewiz/Azenta Life Sciences (Indianapolis, Indiana, USA) was used to confirm mutations. The sequence-confirmed plasmid was transformed into *E. coli* strain BL21 (NEB C2530H) chemically competent cells according to the NEB transformation protocol and plated onto LB plates containing 100 µg ml^−1^ carbenicillin.

To express the HNL6V variant protein, LB medium containing carbenicillin (100 µg ml^−1^, 5 ml) was inoculated with a single bacterial colony from an agar plate and incubated in an orbital shaker at 37°C and 240 rev min^−1^ for 15 h to create a seed culture. A 1 l baffled flask containing Terrific Broth–Amp medium (250 ml) was inoculated with this seed culture. This culture was incubated at 37°C and 240 rev min^−1^ for 3–4 h until the absorbance at 600 nm reached 0.4–1.0 and was then cooled on ice for 30 min. Isopropyl β-d-1-thio­galactopyranoside (0.75–1.0 m*M* final concentration) was added to induce protein expression, and cultivation was continued for 20 h at 18°C. The cells were harvested by centrifugation (4°C, 7000 rev min^−1^, 15 min), resuspended in Ni–NTA loading buffer (10 m*M* imidazole, 50 m*M* Tris pH 8.0, 500 m*M* NaCl, 4 ml per gram of wet cells) and disrupted by sonication (400 W, 40% amplitude for 5 min). The cell lysate was centrifuged to pellet the cell debris (4°C, 12 000 rev min^−1^ for 45 min) and the supernatant was mixed with 1–2.5 ml Ni–NTA resin (pre-equilibrated with 10 ml Ni–NTA loading buffer) and incubated for 45 min at 4°C with rotation (10 rev min^−1^). The resin/supernatant mixture was loaded onto a 25 ml column (Bio-Rad) and the resin was washed with ten column volumes each of buffer containing increasing amounts of imidazole (25–50 m*M* imidazole, 50 m*M* Tris pH 8.0, 500 m*M* NaCl). The His-tagged protein was eluted with ten column volumes of elution buffer and collected in 1 ml fractions (125 m*M* imidazole, 50 m*M* Tris pH 8.0, 500 m*M* NaCl). The protein concentration of each elution fraction was determined from spectrophotometric measurements at 280 nm using a NanoDrop 2000 (Thermo Scientific). The calculated extinction coefficient (45 380 *M*^−1^ cm^−1^) was determined using the *ProtParam* computational web tool (https://web.expasy.org/protparam; Gasteiger *et al.*, 2005 ▸). Protein gels were used to check for the presence and purity of protein and were run using sodium dodecyl sulfate polyacrylamide gradient gels (NuPage 4−12% Bis-Tris gel from Invitrogen) using the Precision Plus Dual Color protein standard (Bio-Rad, 5 µl in each lane) for 50 min at 150 V, stained with SimplyBlue Safe Stain (Thermo Fisher Scientific) and destained twice with Milli-Q UltraPure H_2_O. SDS–PAGE indicated a molecular weight of ∼30 kDa, in agreement with the predicted weight of 31.1 kDa. The imidazole-containing elution buffer was exchanged by the addition of BES buffer [5 m*MN*,*N*-bis(2-hydroxyethyl)-2-amino­ethanesulfonic acid pH 7.2, 14 ml] followed by ultrafiltration (Amicon 15 ml ultrafiltration centrifuge filter, 10 kDa cutoff) to reduce the volume to ∼250 µl. This addition of buffer and filtration was repeated four times. A 250 ml culture yielded 2.2 mg of protein.

### 2.2. Crystallization

**Figure 1:**
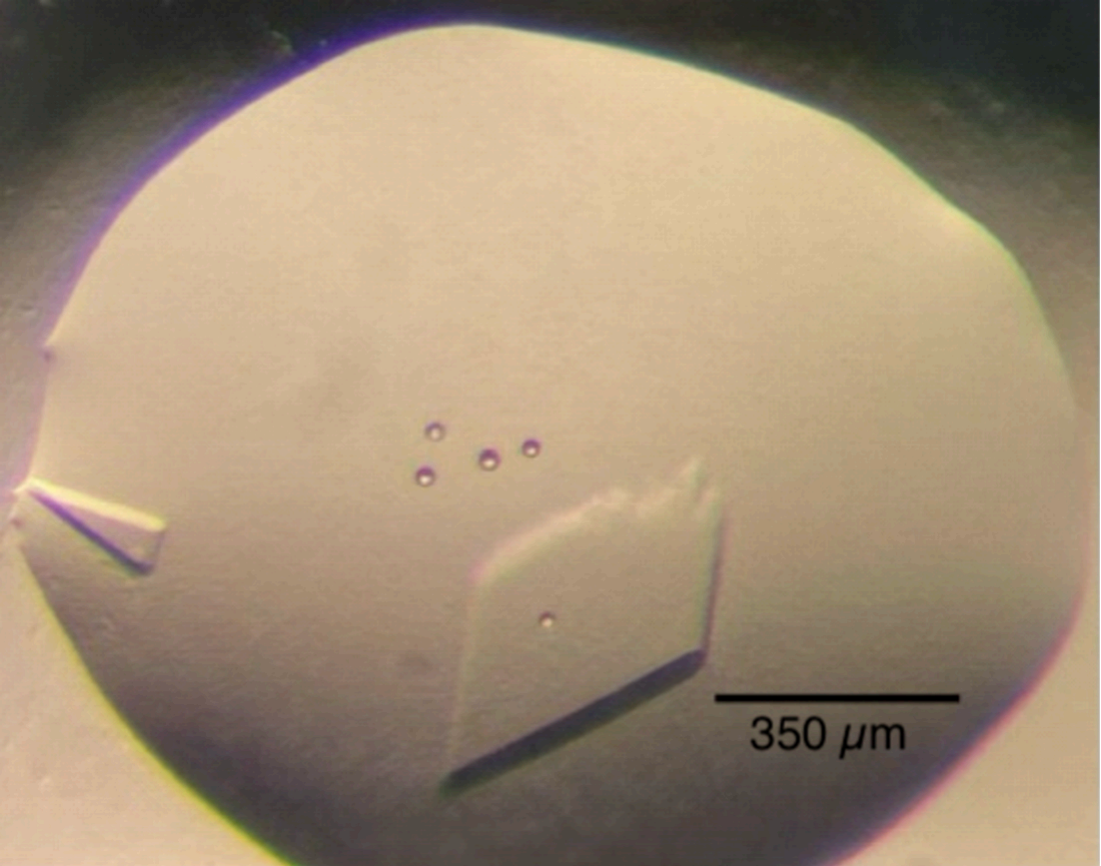
HNL6V crystal used for data collection prior to extraction or soaking. Growth conditions: 0.1 *M* bis-Tris pH 5.5, 2.0 *M* ammonium sulfate. The HNL6V crystals measured 90 × 175 µm (left) and 350 × 300 µm (right). The larger right crystal was harvested for data collection. The five circular ‘bubbles’ are optical artifacts of the microscope.

Crystallographic screening was performed using a Phoenix crystallography dispenser from Art Robbins Instruments. Low-profile Intelli-Plate sitting-drop vapor-diffusion trays from Art Robbins Instruments were used for crystallization setup. All of the setup and washing procedures were performed through the Art Robbins Instruments Phoenix software. Each crystallization drop contained 0.1 µl protein sample (9.3 mg ml^−1^ protein) and 0.1 µl well solution. A total of 960 conditions were tested. Crystals appeared within one day from the Index HT screen from Hampton Research under the condition 0.1 *M* bis-Tris pH 5.5, 2 *M* ammonium sulfate and grew to a full size of 0.35 mm in three days (Fig. 1 ▸, Table 2 ▸). The data from the crystals obtained by robotic screening proved sufficient to refine the model so additional crystal screening trays were not needed.

### 2.3. Data collection, processing and structure refinement

Crystals were transferred into cryoprotectant solutions consisting of well-solution components and increasing concentrations of sodium malonate. The final concentration of sodium malonate in the cryoprotectant solution was 1.2 *M*. Harvested crystals were flash-cooled in liquid nitrogen. The structural data set was collected on beamline 24-ID-C (NE-CAT) at the Advanced Photon Source (APS), Argonne National Laboratory at an oscillation angle of 0.2°. Reconstructed ancestral hydroxynitrile lyase (Jones *et al.*, 2020 ▸; PDB entry 5tdx) with 76% sequence identity was used for molecular replacement and refined to a 2.3 Å resolution model. This 2.3 Å resolution initial model was used to aid in the refinement of the second and final model, resulting in a 1.99 Å resolution structure. *HKL*-2000 (Otwinowski & Minor, 1997 ▸) was used to process the collected data and *Phaser* (McCoy *et al.*, 2007 ▸) in *Phenix* (Liebschner *et al.*, 2019 ▸) was used for molecular replacement and refinement. Refinement modeling was performed using *Coot* (Emsley *et al.*, 2010 ▸). During structural refinement, electron density was observed in the active site, but no ligand was found to sufficiently fulfill the electron density (Supplementary Fig. S2). The final data-collection and refinement statistics are shown in Tables 3 ▸ and 4 ▸, respectively. The final model was deposited in the Research Collaboratory for Structural Bioinformatics Protein Data Bank (PDB entry 8euo).

The Protein Data Bank contains 18 structures of wild-type hydroxynitrile lyase from *H. brasiliensis*, which were used for comparison: PDB entries 1sci, 1sck, 1scq, 1sc9 (Gruber *et al.*, 2004 ▸), 1yas (Wagner *et al.*, 1996 ▸), 2yas, 3yas, 4yas, 5yas, 6yas, 7yas (Zuegg *et al.*, 1999 ▸), 1yb6, 1yb7 (Gartler *et al.*, 2007 ▸), 3c70, 3c6x, 3c6y, 3c6z (Schmidt *et al.*, 2008 ▸) and 1qj4 (Gruber *et al.*, 1999 ▸). The three structures of salicylic acid-binding protein 2 from tobacco used for comparison have the following PDB codes: 1y7i, 1y7h and 1xkl (Forouhar *et al.*, 2001 ▸). *PyMOL* v.2.5.4 and v.3.0 were used to create images of protein structures (Schrödinger; https://pymolwiki.org). The supporting information contains an example *PyMOL* script to align the structures using the *align* function and measure distances between corresponding C^α^ atoms in the HNL6V structure and 18 *Hb*HNL structures. The statistical significance of the mean distance between C^α^ atoms in HNL6V and the 18 *Hb*HNL structures and the mean distance between C^α^ atoms in one *Hb*HNL structure with the other 17 *Hb*HNL structures (all 153 possible pairwise combinations) was analyzed for significance using a two-tailed *t*-test with unequal variances (Welch’s *t*-test) using equation (1). The *t*-test, degrees of freedom and *p*-value were calculated using a web tool (https://www.statskingdom.com/150MeanT2uneq.html).
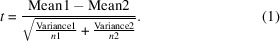


## 3. Results and discussion

### 3.1. Crystallization and structure determination

HNL6V containing a C-terminal His_6_-tag was expressed from a pET-21a(+) plasmid containing the gene for HNL6V in *E. coli* BL21. The protein was purified using nickel-affinity chromatography and concentrated to 9.3 mg ml^−1^. Screening of crystallization conditions yielded initial crystals within one day and full-size crystals within three days (Fig. 1 ▸). The harvested crystal belonged to space group *C*222_1_, with unit-cell parameters *a* = 47.05, *b* = 106.38, *c* = 128.40 Å and one molecule per asymmetric unit. The structure of a reconstructed ancestral hydroxynitrile lyase (Jones *et al.*, 2020 ▸) with 76% sequence identity was used for molecular replacement. The model was refined to 1.99 Å resolution, with *R*_work_ and *R*_free_ values of 0.1844 and 0.2376, respectively (Table 4 ▸).

Structural refinement revealed 2*F*_o_ − *F*_c_ (blue) and *F*_o_ − *F*_c_ (red/green) electron density in the active site which could not be assigned to a ligand (Supplementary Fig. S2). Placing water, glycerol, malonate or sulfate in various orientations within this region did not improve the *R*_work_ and *R*_free_ statistics, nor did these placements satisfy the density. As this electron density near the catalytic serine O^γ^ remains unmodeled, this model should be considered a putative structure. Zuegg *et al.* (1999 ▸) also observed unidentified electron density near the active site during their refinement of an X-ray crystal structure of wild-type *Hb*HNL.

### 3.2. Structural analysis

**Figure 2:**
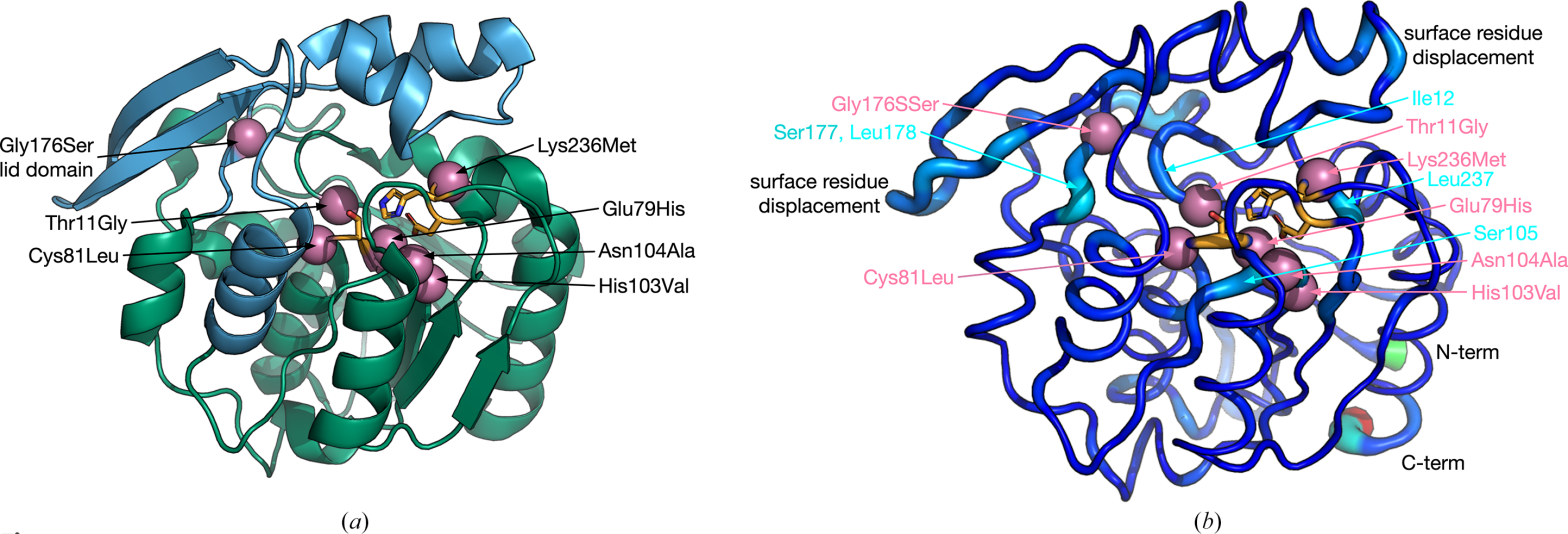
Locations of substitutions and displacements of C^α^ positions in HN6V (PDB entry 8euo) relative to wild-type HNL from *H. brasiliensis*. Both figures show the catalytic triad (Ser80, His235 and Asp207) in orange sticks with the O^γ^ atom of Ser80 in red at the center of the figure. The pink spheres show the C^α^ atoms of the seven substitutions (Thr11Gly, Glu79His, Cys81Leu, His103Val, Asn104Ala, Gly176Ser and Lys236Met). (*a*) Ribbon diagram of the X-ray structure of HNL6V (PDB entry 8euo) showing the catalytic domain (α/β-hydrolase fold) in green and the lid domain in blue (residues 115–178). Substitution Gly176Ser is in the lid domain, while the other six are in the catalytic domain. (*b*) Displacement of C^α^ positions in HNL6V compared with *Hb*HNL (PDB entry 3c6x). The larger diameter and warmer colors in the cartoon representation indicate larger C^α^ displacements between structures. The large displacements at the N- and C-termini and the smaller surface-residue displacements are likely to be due to differences in crystallization contacts and unrelated to the substitutions. Among the seven substitutions, only His103Val and Asn104Ala exhibit significant displacement, along with the neighboring residue Ser105. The remaining five substitutions maintain their C^α^ positions but induce conformational changes in adjacent residues (labeled in turquoise).

HNL6V adopts the same protein fold as wild-type *Hb*HNL. The catalytic domain follows the α/β-hydrolase fold and contains the catalytic triad and oxyanion-hole residues (Fig. 2 ▸*a*). The lid or cap domain (residues 115–178; Wagner *et al.*, 1996 ▸) covers the active site to create a substrate-binding pocket. The seven amino-acid substitutions in HNL6V were in and around the active site. Six of the substitutions are in the catalytic domain; only Gly176Ser is in the lid domain. None of the substitutions were on the protein surface.

The distances between corresponding atom positions in HNL6V and those in *Hb*HNL or SABP2 are typically <1 Å. The statistical significance of these differences relies on a two-tailed *t*-test with unequal variance (Welch’s *t*-test), which relies on the number of independent comparisons. The Protein Data Bank contains 18 independent structures of *Hb*HNL, but only three independent structures of SABP2. Because of this difference in the number of comparison structures, the comparisons between HNL6V and *Hb*HNL are often statistically significant, while those between HNL6V and SABP2 are rarely statistically significant.

The C^α^ positions in HNL6V differ from those in the parent *Hb*HNL. There are 18 structures of wild-type HNL from *H. brasiliensis* available in the Protein Data Bank. Pairwise superposition of these structures with each other (153 pairs) using the *align* function of *PyMOL* yielded a mean distance of 0.13 ± 0.09 Å between the corresponding C^α^ atoms. In contrast, pairwise superposition of HNL6V onto these 18 wild-type structures yielded a larger mean distance of 0.2 ± 0.1 Å between corresponding C^α^ atoms. (The supplementary information includes a *PyMOL* script for this calculation.) The difference between HNL6V–*Hb*HNL mean distances and *Hb*HNL–*Hb*HNL mean distances is significant (two-tailed *p*-value = 0.01) using a two-sample *t*-test with unequal variances. Thus, the seven amino-acid substitutions in HNL6V changed the backbone positions compared with wild-type *Hb*HNL.

The C^α^ positions in HNL6V do not differ from those in the target SABP2 at a statistically significant level. Pairwise superposition of the three SABP2 structures with each other (three pairs) yielded a mean distance of 0.21 ± 0.07 Å between the corresponding C^α^ atoms. Pairwise superposition of HNL6V onto these three structures yielded a larger mean distance of 0.6 ± 0.3 Å between corresponding C^α^ atoms. In spite of this larger distance, the difference between HNL6V–SABP2 mean distances and SABP2–SABP2 mean distances is not significant (two-tailed *p*-value = 0.14) using a two-sample *t*-test with unequal variances. This uncertain result is partly due to the lower statistical power when comparing only three SABP2 structures, compared with the 18 *Hb*HNL structures above.

**Figure 3:**
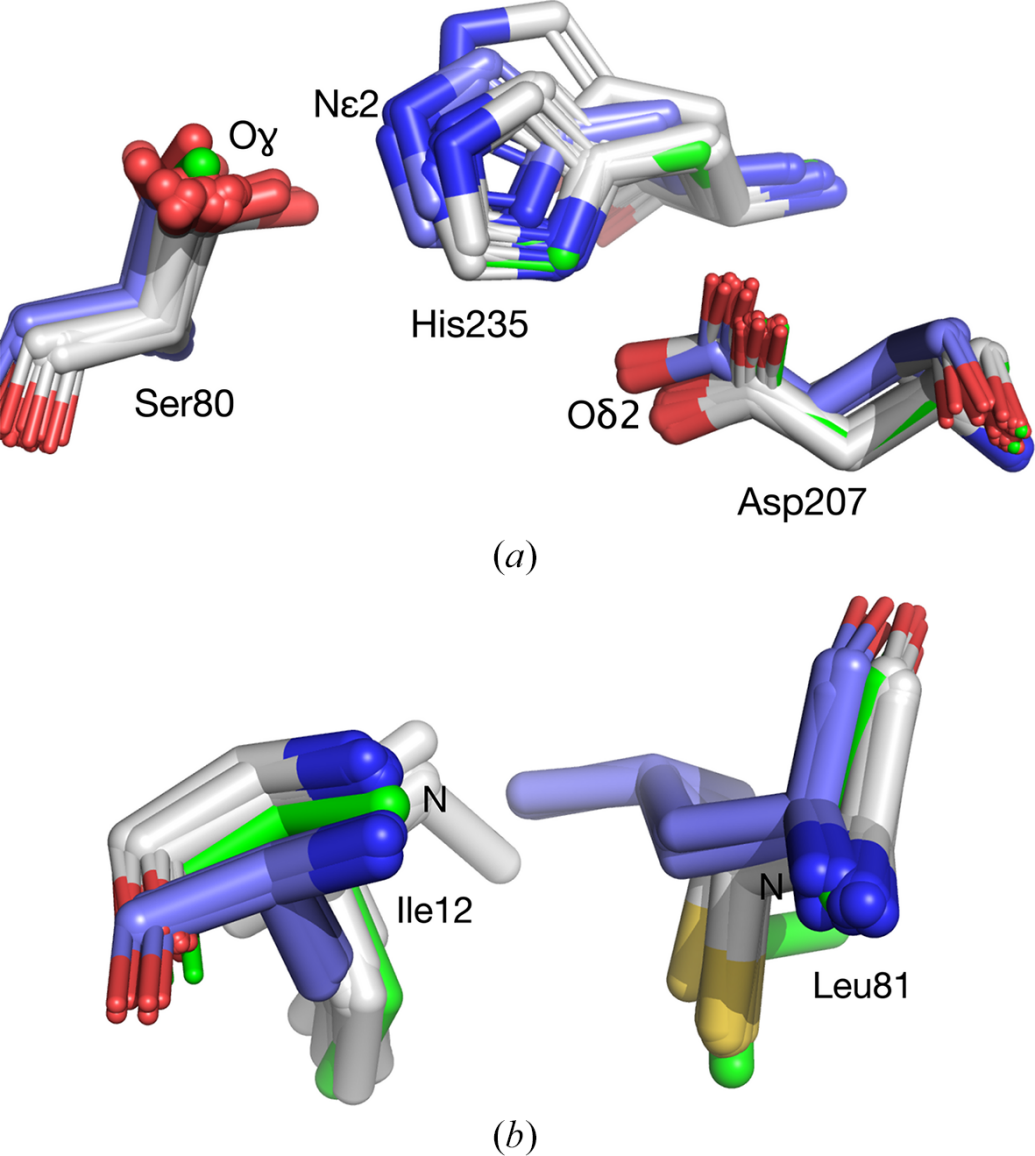
Best-fit overlay of the C^α^ positions of SABP2 structures (three structures, light blue C atoms) and *Hb*HNL structures (18 structures, white C atoms) onto the structure of HNL6V (green sticks). (*a*) The catalytic atoms of the catalytic triad in HNL6V (O^γ^ of Ser80, N^ɛ2^ of His235, O^δ2^ of Asp207) overlay more closely with the corresponding atoms in the *Hb*HNL structures (Ser80, His235 and Asp207) than with the corresponding atoms in the SABP2 structures (Ser81, His238 and Asp210). (*b*) The oxyanion-hole amide N atoms of Ile12 and Leu81 in HNL6V overlay more closely with the corresponding atoms in *Hb*HNL (Ile12 and Cys81) than with the corresponding atoms in SABP2 (Ala13 and Leu82). The average distances are listed in Table 5 ▸. The side-chain conformations differ at OX2 (Cys81 in *Hb*HNL, Leu82 in SABP2) and HNL6V maintains an *Hb*HNL-like conformation despite the Cys81Leu substitution.

The five catalytic atom positions in HNL6V are all closer to those in *Hb*HNL than to those in SABP2 (Table 5 ▸), but they lie between the two average positions of the wild-type proteins (Fig. 3 ▸). The differences are statistically significant for the Asp O^δ2^ and His N^ɛ2^ positions, but not for the other three, mainly because of the fewer SABP2 comparison structures. This shift shows that the substitutions have made the catalytic atom positions in HNL6V closer to those in SABP2, but they still resemble the catalytic positions of *Hb*HNL more closely than those of SABP2.

The side-chain conformation differs at OX2 in SABP2 and *Hb*HNL, and HNL6V maintains the *Hb*HNL-like conformation. In SABP2 the side chain of Leu82 points away from the region where the oxyanion is expected to bind, but in *Hb*HNL the side chain of Cys81 points towards this region (Fig. 3 ▸*b*). Although HNL6V contains the Cys81Leu substitution, the side chain of the leucine still points in the same direction as the cysteine in *Hb*HNL.

The seven substitutions in HNL6V change the C^α^ positions at at the substitution sites and at some adjacent residues. Fig. 2 ▸(*b*) and Table 6 ▸ compare a single high-resolution (1.05 Å) wild-type *Hb*HNL structure (PDB entry 3c6x) with HNL6V. The mean C^α^ displacement (0.20 ± 0.18 Å over 244 of 257 atoms) is similar to the mean for the comparison of all 18 structures. Some differences between the two structures are likely to be due to different crystallization conditions rather than the engineered mutations: large deviations at the N-terminus (Phe3, ΔC^α^ = 0.96 Å) and C-terminus (Tyr256, ΔC^α^ = 0.62 Å; Asn257, ΔC^α^ = 2.4 Å), as well as smaller differences at various positions on the protein surface. Among the seven amino-acid substitutions, the C^α^ positions at Asn104Ala and His103Val shift moderately (0.50 and 0.37 Å, respectively, similar to that in the mean values above), with the adjacent residue Ser105 also shifting (0.41 Å). The Gly176Ser mutation shows minimal displacement at the substitution site (0.23 Å), but the C^α^ positions at the adjacent residues Ser177 and Leu178 change by a larger amount (0.39 and 0.49 Å, respectively). Similarly, Lys236Met causes an average displacement at the substitution site (0.23 Å), while the C^α^ atom at the adjacent Leu237 moves twice as much (0.46 Å). The remaining substitutions (Thr11Gly, Glu79His and Cys81Leu) show average or smaller displacements at their respective positions (0.23, 0.25 and 0.09 Å, respectively), but also cause displacements nearby. Ile12 (an oxyanion-hole residue next to Thr11Gly) and Ser80 (the catalytic serine next to Cys81Leu) both move by 0.33 Å. In addition, Gly83 on the subsequent helical turn to Cys81Leu also moves by 0.35 Å.

To identify whether the substitutions in HNL6V altered the flexibility of some regions of the protein more than others, we compared the normalized *B* factors at the C^α^ atoms in HNL6V with each of the 18 comparison structures. The *B* factors in the HNL6V structure (mean of 27.1 Å^2^) are higher than they are in the 18 comparison *Hb*HNL structures (mean of 12.7 Å^2^). This difference is likely to be due to the different experimental conditions associated with solving the different structures. To compare the *B* factors between proteins, we normalized the *B* factors to *Z*-scores. A *Z*-score of zero corresponds to an average *B* factor for that protein, while a value of 1 corresponds to a *B* factor one standard deviation higher than average. Thus, this normalization identifies regions that are more or less flexible than average.

**Figure 4:**
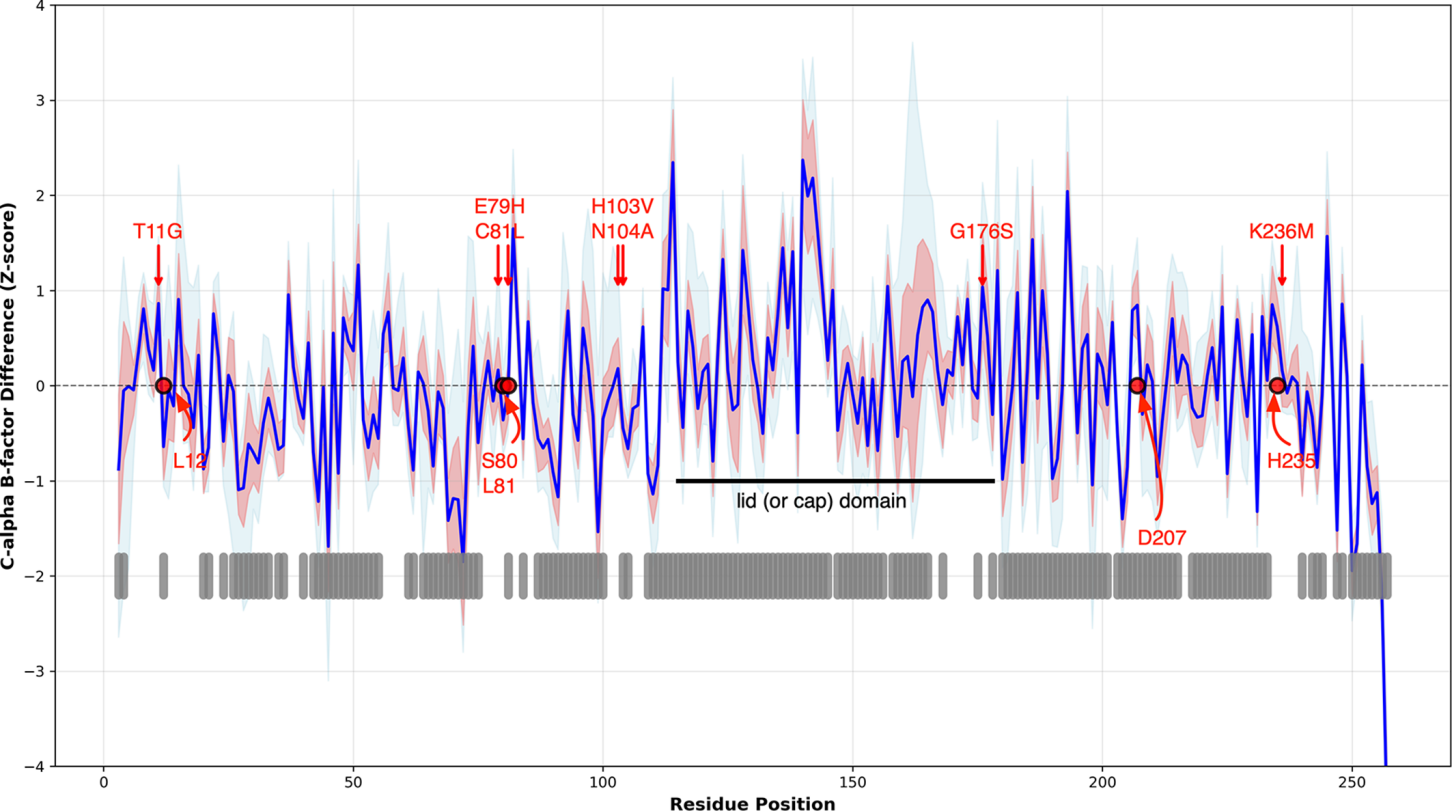
The largest increases in *B* factors (normalized as *Z*-scores) for the C^α^ atoms in HNL6V compared with 18 wild-type *Hb*HNL structures occur in the lid domain (residues 108–179, marked by a black horizontal line at *Z*-score = −1). Changes occur mostly among the 186 surface residues (thick gray vertical lines below the plotted data). The blue line shows the mean difference accross 18 pairwise comparisons of the HNL6V structure with one of the 18 wild-type *Hb*HNL structures. The light red area shows ±1 standard deviation of the differences and the light blue area shows the full range (min to max). The red arrows mark the substitution positions and the red circles mark the catalytic triad and oxyanion-hole residues.

The average difference in *Z*-score at each C^α^ position between HNL6V and each of the 18 comparison wild-type *Hb*HNL structures (Fig. 4 ▸) is −0.02σ ± 0.8. A value near zero is expected since the *Z*-score normalized the *B* factors for each protein to an average value of zero. Differences in *B* factors are mostly small (less than ±1σ) and occur throughout the protein, but especially at the 186 residues that are exposed to the solvent. (The 70 buried residues were defined as those having less than 23 Å^2^ solvent-exposed area.) The largest changes occur in the lid (or cap) domain (residues 115–178). The seven substitution positions showed slightly higher than average *B*-factor differences of +0.3σ ± 0.5.

Among the five catalytic amino-acid residues, Asp207 and His235 in the catalytic triad show higher relative *B* factors by almost one standard deviation (+0.8σ ± 0.4 and +0.6σ ± 0.3, respectivly) and the oxyanion residue Leu12 and the catalytic serine 80 show lower relative *B* factors by about half of a standard deviation (−0.6σ ± 0.3 and −0.4σ ± 0.3, respectively). The oxyanion-hole residue Leu81 shows little change (−0.1σ ± 0.3). Substitutions in HNL6V primarily increase flexibility in the lid domain and at specific catalytic residues, with the overall *B*-factor differences remaining small and concentrated in surface-exposed regions.

## 4. Conclusions

The crystal structure of HNL6V reveals that seven amino-acid substitutions, modeled after residues in the esterase SABP2 and located within and outside the substrate-binding site, induce modest but significant shifts in backbone positions and catalytic atom locations relative to wild-type *Hb*HNL, moving them closer to those of SABP2 while preserving the overall α/β-hydrolase fold. These substitutions also increase local flexibility, particularly in the lid domain and at certain catalytic residues, although overall structural changes remain small.

## 5. Related literature

The following references are cited in the supporting information for this article: Madeira *et al.* (2024 ▸) and Sievers *et al.* (2011 ▸).

## Supplementary Material

PDB reference: hydroxynitrile lyase from *Hevea brasiliensis* with seven mutations, 8euo

Supplementary Figures and PyMOL script. DOI: 10.1107/S2053230X25007034/ft5125sup1.pdf

## Figures and Tables

**Table 1:** Macromolecule-production information

Source organism	*Hevea brasiliensis*
DNA source	Hasslacher *et al.* (1996 ▸)
Forward primers	
C81L	CAGCCTGGGAGGACTCAATATAGCAATTG
N104A	TGTTTTCGTCGCGTCAGTATTGCCAGACAC
G176S	GACAAGGAAGAGCTCATTATTTCAAAATATTTTAGC
Reverse primers	
C81L	TGGCCAACCAGAATCACCTTTTCCCC
N104A	GCAGCTGCAATCTTTTCAC
G176S	AACATCTTCGCCAGTTCATATTC
Expression vector	pET-21a(+)
Expression host	*E. coli* BL21
Complete amino-acid sequence of the construct produced	MAFAHFVLIHGICHGAWIWHKLKPLLEALGHKVTALDLAASGVDPRQIEEIGSFDEYSEPLLTFLEALPPGEKVILVGHSLGGLNIAIAADKYCEKIAAAVFVASVLPDTEHCPSYVVDKLMEVFPDWKDTTYFTYTKDGKEITGLKLGFTLLRENLYTLCGPEEYELAKMLTRKSSLFQNILAKRPFFTKEGYGSIKKIYVWTDQDEIFLPEFQLWQIENYKPDKVYKVEGGDHMLQLTKTKEIAEILQEVADTYNLEHHHHHH

**Table 2:** Crystallization

Method	Vapor diffusion, sitting drop
Plate type	CrystalMation Intelli-Plate 96-3 low-profile
Temperature (K)	293
Protein concentration (mg ml^−1^)	9.3
Buffer composition of protein solution	5 m*M* BES pH 7.2
Composition of reservoir solution	0.1 *M* bis-Tris pH 5.5, 2 *M* ammonium sulfate
Volume and ratio of drop (µl)	200, 1:1 (protein:screen solution)
Volume of reservoir (µl)	50

**Table 3:** Data collection and processing Values in parentheses are for the outer shell.

Diffraction source	24-ID-C, APS
Wavelength (Å)	0.979
Detector	Dectris EIGER2 S 16M
Crystal-to-detector distance (cm)	230
Rotation range per image (°)	0.2
Exposure time per image (s)	0.2
Space group	*C*222_1_
*a*, *b*, *c* (Å)	47.05, 106.38, 128.40
α, β, γ (°)	90, 90, 90
Mosaicity (°)	0.2
Resolution range (Å)	43.03–1.99 (2.04–1.99)
Total No. of reflections	102923 (7877)
No. of unique reflections	20181 (1606)
Completeness (%)	89.43 (89,95)
Multiplicity	5.1
〈*I*/σ(*I*)〉	13.9 (2.3)
*R*_r.i.m._	0.032 (0.490)
Overall *B* factor from Wilson plot (Å^2^)	28.09

**Table 4:** Structure solution and refinement Values in parentheses are for the outer shell.

No. of reflections, working set	20178 (2005)
No. of reflections, test set	2000 (199)
Final *R*_work_	0.1849 (0.2285)
Final *R*_free_	0.2374 (0.2603)
No. of non-H atoms	
Protein	2030
Ligand	0
Solvent	123
Total	2162
R.m.s. deviations	
Bonds (Å)	0.009
Angles (°)	1.03
Ramachandran plot	
Favored (%)	96.44
Allowed (%)	3.56
Outliers (%)	0.0

**Table 5:** Comparison of the structure of HNL6V with 18 *Hb*HNL structures and three SABP2 structures

Comparison atoms	Distance between three SABP2 structures (Å)	Distance between HNL6V and *Hb*HNL structures (Å)	Distance between HNL6V and SABP2 structures (Å)	Statistically significant†
All C^α^		0.2 ± 0.1	0.6 ± 0.3	No
Ser O^γ^	1.6 ± 0.6	0.8 ± 0.4	1.1 ± 0.8	No
His N^ɛ2^	0.31 ± 0.1	0.3 ± 0.3	0.77 ± 0.07	Highly significant
Asp O^δ2^	0.25 ± 0.07	0.2 ± 0.1	0.77 ± 0.05	Highly significant
OX1 N	0.25 ± 0.03	0.43 ± 0.09	0.9 ± 0.2	No
OX2 N	0.33 ± 0.06	0.21 ± 0.07	0.26 ± 0.09	No

† Statistically significant differences between HNL6V–*Hb*HNL differences and HNL6V–SABP2 differences.

**Table 6:** Change in C^α^ positions at the substitution location and at nearby residues Comparison between HNL6V (PDB entry 8euo) and *Hb*HNL (PDB entry 3c6x). Larger than average changes are in bold.

Substitution	Displacement at substitution site (Å)	Displacement at adjacent residues (Å)
Thr11Gly	0.23	Ile12 (OX1), **0.33**
Glu79His	0.25	—
Cys81Leu	0.09	Ser80 (catalytic), **0.33**; Gly83, **0.35**
His103Val	**0.37**	—
Asn104Ala	**0.50**	Ser105, **0.41**
Gly176Ser	0.23	Ser177, **0.39**; Leu178, **0.49**
Lys236Met	0.23	Leu237, **0.46**
