# Supplementary material for: Crystal structure of a seven-substitution mutant of hydroxynitrile lyase from rubber tree
Source: Acta Crystallogr F Struct Biol Commun. 2025 Aug 27;81(Pt 9):398–405. doi: 10.1107/S2053230X25007034 (PMC12400193; doi:10.1107/S2053230X25007034)
Supplement: Supplementary file 1 [file f-81-00398-sup1.pdf]

Acta Cryst F
STRUCTURAL BIOLOGY
COMMUNICATIONS

Volume 81 (2025)

Supporting information for article:

Crystal structure of a seven-substitution mutant of hydroxynitrile lyase from rubber tree

Colin T. Pierce, Lauren R. Greenberg, Meghan E. Walsh, Ke Shi, Drenen J. Magee, Hideki Aihara, Wendy Gordon, Robert L. Evans and Romas J. Kazlauskas

```
SABP2      MKEGKHFVLVHGACHGGWSWYKLKPLLEAAGHKVTALDLAASGTDLRKIEELRTLYDYTL 60
HNL6V      -MAFAHFVLIHGICHGAWIWHKLKPLLEALGHKVTALDLAASGVDPRQIEEIGSFDEYSE 59
HbHNL      -MAFAHFVLIHTICHGAWIWHKLKPLLEALGHKVTALDLAASGVDPRQIEEIGSFDEYSE 59
                ****:*  ***.* *:******** *************.* *:***: :: :*:

SABP2      PLMELMESLSADEKVILVGHSLGGMNLGLAMEKYPQKIYAAVFLAAFMPDSVHNSSFVLE 120
HNL6V      PLLTFLEALPPGEKVILVGHSLGGLNIAIAADKYCEKIAAAVFVASVLPDTEHCPSYVVD 119
HbHNL      PLLTFLEALPPGEKVILVGESCGGLNIAIAADKYCEKIAAAVFHNSVLPDTEHCPSYVVD 119
           **: ::*:*  .*******.* **:*:.:* :** :** ****  :.:**: *  *:*::

SABP2      QYNERTPAENWLDTQFLPYGSPEEPLTSMFFGPKFLAHKLYQLCSPEDLALASSLVRPSS 180
HNL6V      KLMEVF--PDWKDTTYFTYTKDGKEITGLKLGFTLLRENLYTLCGPEEYELAKMLTRKSS 177
HbHNL      KLMEVF--PDWKDTTYFTYTKDGKEITGLKLGFTLLRENLYTLCGPEEYELAKMLTRKGS 177
           :  *     :* ** :: * .  : :*.: :* .:* .:** **.**:  **. *.* .*

SABP2      LFMEDLSKAKYFTDERFGSVKRVYIVCTEDKGIPEEFQRWQIDNIGVTEAIEIKGADHMA 240
HNL6V      LFQNILAKRPFFTKEGYGSIKKIYVWTDQDEIFLPEFQLWQIENYKPDKVYKVEGGDHML 237
HbHNL      LFQNILAKRPFFTKEGYGSIKKIYVWTDQDEIFLPEFQLWQIENYKPDKVYKVEGGDHKL 237
           ** : *:*  :**.* :**:*::*:   :*: :  *** ***:*    :. :::*.**

SABP2      MLCEPQKLCASLLEIAHKYN     260
HNL6V      QLTKTKEIAEILQEVADTYN     257
HbHNL      QLTKTKEIAEILQEVADTYN     257
            * : :::.  * *:*..**
```

Figure S1 Sequence alignment of HNL6V to the starting sequence *Hb*HNL and the target sequence SABP2. The substitutions in HNL6V are marked with a box. The colors indicate the physiochemical properties of the amino acid. Amino acids conserved across all three sequences are marked with ‘*’, those with strongly similar properties are marked with ‘:’, those with weakly similar properties are marked with ‘.’ and those with no consevation are marked with a blank. Sequences were aligned with Clustal Omega (Sievers *et al.*, 2011) as implemented by the EMBL-EBI Job Dispatcher web tool (Madiera *et al.*, 2024). HNL6V also contained a C-terminal His_6-tag which is not shown in this alignment.

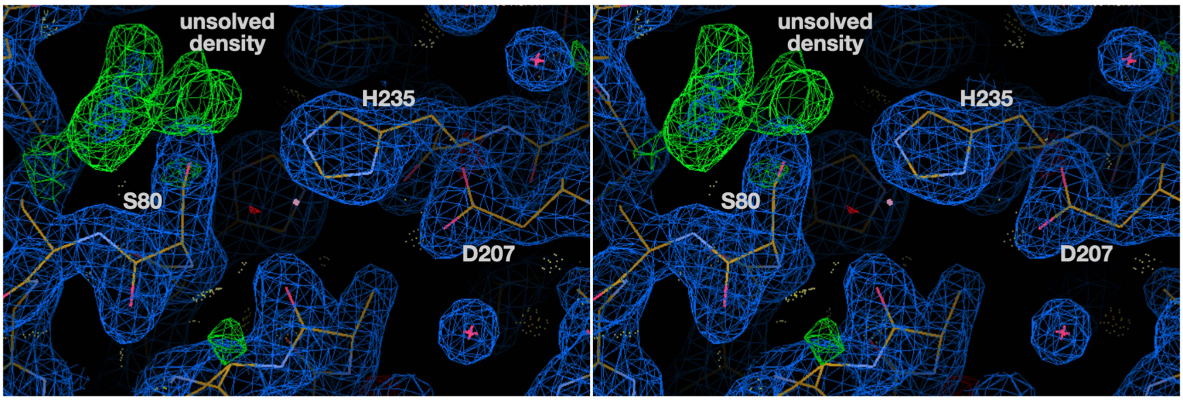

Figure S2 Cross-eyed stereo view of the unmodeled density (green mesh) of HNL6V near the active site at at a 3.0 σ contour level. The electron density associated with the catalytic triad (S80-D207-H235) is labeled.

S1. Pymol script to compare Cα distances between 8EUO and the corresponding Cα in a structure of *Hb*HNL

```
# Compares the distances between Calphas in one protein (8EUO)
# to those in one of eighteen homologs. Downloads the needed pdb
# files to the Desktop and prints results to the PyMOL command
# line window. The script takes ~5 min to complete.
#
# Instructions:
# 1. save this script as a text file with the extension .pml
# 2. open PyMOL
# 3. drag this file onto the PyMOL viewing window to run the
# commands below
#
# Romas Kazlauskas, January, 2023

# clear any settings
reinitialize

# set working directory to Desktop
```

```
cd ~/Desktop

# download HNL6V structure from from the PDB to the working directory
fetch 8EUO, type = pdb

# list of structures to compare to 8EUO
homolog = ["1SCI", "1YAS", "2YAS", "3YAS", "4YAS", "5YAS", "6YAS", "7YAS",
"1YB6", "1YB7", "1SCK", "1SCQ", "1SC9", "3C70", "3C6X", "3C6Y", "3C6Z",
"1QJ4"]

# open pdb files and align homologs to 8EUO
for item in homolog: cmd.fetch(item, type = 'pdb')
for item in homolog: cmd.align(item, "8EUO")

# remove non-protein atoms and all except chain A
remove not polymer.protein or not chain A

# python section below is needed to run loops that require more than one
line
python

# used to calculate standard deviation
import statistics

# create a list to store distances for all homologs
all_distances = []

# loop over all homologs
for j in range(len(homolog)):

# Create a list to store the distances for each homolog
```

```
        distances = []

# measure the distances between Calphas in 8EUO and the current homolog
        for i in range (3,257):
                distance = cmd.distance("(/"+homolog[j]+"///"+str(i)
                +"/CA)","(/8EUO///"+str(i)+"/CA)")
                distances.append(distance)

# Calculate the mean & standard deviation of the distances
        mean = sum(distances) / len(distances)
        std_dev = statistics.stdev(distances)

# Print the results for the comparison to the current homolog
        print(homolog[j], "- 8EUO", len(distances),"distances, ",
        "%8.3f"%mean, "±", "%8.3f"%std_dev)

# add the list of distances for this homolog to the list of all distances
        all_distances.extend(distances)

# increment counter to move to the next homolog
        j += 1
python end

# Calculate and print results for all eighteen comparisons combined
all_mean = sum(all_distances) / len(all_distances)
all_std_dev = statistics.stdev(all_distances)
print(len(all_distances),"combined distances", "%8.3f"%all_mean, "±",
"%8.3f"%all_std_dev)
```

References

Madeira, F., Madhusoodanan, N., Lee, J., Eusebi, A., Niewielska, A., Tivey, A. R. N., Lopez, R. & Butcher, S. (2024). *Nucleic Acids Res.* **52**, W521—W525.

Sievers, F., Wilm, A., Dineen, D., Gibson, T. J., Karplus, K., Li, W., Lopez, R., McWilliam, H., Remmert, M., Söding, J., Thompson, J. D. & Higgins, D. G. (2011). *Mol. Syst. Biol.*, **7**, 539.
